# What is the current evidence base for measles vaccination earlier than 9 months of age?: Report from an informal technical consultation of the World Health Organization

**DOI:** 10.1016/j.vaccine.2025.127187

**Published:** 2025-05-31

**Authors:** Anshu Varma, Shelly Bolotin, Gaston De Serres, Arnaud M. Didierlaurent, Kristen Earle, Kurt Frey, Susan Hahné, Daniel Kapelus, L. Kendall Krause, Kevin McCarthy, William J. Moss, Walter A. Orenstein, Rob van Binnendijk, Dorthe Maria Vittrup, Merryn Voysey, Tom Woudenberg, Naor Bar-Zeev, Anindya S. Bose, Joachim Hombach, Mick N. Mulders, Laura Nic Lochlainn, Kezia Suwintono, Daniel R. Feikin, Natasha S. Crowcroft

**Affiliations:** a Department of Immunization, Vaccines, and Biologicals World Health Organization, Geneva, Switzerland; b Centre for Vaccine Preventable Diseases, Dalla Lana School of Public Health, and Department of Laboratory Medicine and Pathobiology, University of Toronto, Toronto, Canada; c Faculty of Medicine, Laval University, Quebec, Canada; d Center of vaccinology, Department of Pathology and Immunology, University of Geneva, Geneva, Switzerland; e Vaccine Development, Bill & Melinda Gates Foundation, Washington, United States; f Institute for Disease Modelling, Bill & Melinda Gates Foundation, Washington, United States; g Center for Infectious Disease Control, National Institute for Public Health and the Environment, Bilthoven, the Netherlands; h Wits Vaccines and Infectious Diseases Analytics Research Unit, University of Witwatersrand, Johannesburg, South Africa; i Immunization, Bill & Melinda Gates Foundation, Washington, United States; j International Vaccine Access Center, Johns Hopkins Bloomberg School of Public Health, Baltimore, MD, United States; k Emory Vaccine Center, Emory University, Atlanta, United States; l Child and adolescent Department, Rigshospitalet, Copenhagen, Denmark; m Oxford Vaccine Group, Department of Pediatrics, University of Oxford, Oxford, United Kingdom

**Keywords:** Early measles vaccination, Immune blunting, Vaccine effectiveness, Immunogenicity

## Abstract

Measles is one of the most contagious vaccine preventable diseases, causing severe complications and deaths globally. While vaccination with a measles-containing vaccine (MCV) has prevented millions of measles deaths, recent trends, especially from low- and middle-income countries, are discouraging. Measles cases have increased since 2021 as MCV coverage has decreased; and an estimated 107,500 measles deaths, mostly in children under-five years, occurred in 2023. Thus, a renewed focus on proven and innovative strategies to control measles is needed. The World Health Organization (WHO) recommends a first MCV dose administered at 9–15 months of age (routine MCV1), however MCV1 below 9 months of age (early MCV1) may increase vaccination coverage because uptake of all vaccines tends to be higher the younger the child, and this might protect vulnerable infants earlier in life. However, due to concerns about possible reduced vaccine performance, early MCV1 is not routinely recommended by WHO. WHO hosted an informal technical consultation on December 6–7, 2023, in Geneva, Switzerland to evaluate recent evidence on early MCV1 and identify evidence gaps for policy making. The recent evidence suggests a robust humoral immune response shortly after early MCV1 at 5–8 months of age. Immune blunting of a routine second MCV dose (e.g., MCV2) after early MCV1 was not demonstrated in the presented data. However, 3–7 years after MCV1, children receiving early MCV1 had lower measles antibodies than children receiving routine MCV1, suggesting faster waning of immunity. The totality of evidence on immune blunting remains inconsistent. Meeting participants thought more data are needed before revisiting WHO's current recommendation for a potential revision. Evidence gaps include: understanding measles disease burden and severity in infants; early MCV1 effectiveness and duration; vaccine-induced cellular immunogenicity; whether measles in infants is acquired from other infants or older children or adults; and blunting of routine MCV2. Addressing evidence gaps through targeted studies and measles outbreak investigations, as well as evaluations of country-level introductions of early MCV1 are warranted. Ensuring high MCV1 and MCV2 coverage remains the priority in measles control.

## Highlights

• WHO recommends a first measles vaccine at 9-15 months; an earlier dose may increase coverage and protect younger infants.
• WHO hosted a meeting to evaluate new evidence on an early measles dose and identify evidence gaps for future policy.
• A robust antibody response occurred after a dose at 5-8 months, but may wane faster than after a first dose at the standard age.
• The data did not raise concerns about immune blunting of a second dose.
• New data does not support a change in the current WHO recommendation for a first dose at 9-15 months of age.
• Evidence gaps are severe measles burden and transmission in young infants, vaccine effectiveness, cellular immunogenicity, duration of protection of an early dose.

## 1 Background and meeting objectives

Measles is one of the most contagious vaccine preventable diseases globally and leads to severe complications and deaths, especially in young children from low- and middle-income countries (LMICs) [1]. An estimated cumulative total of 60 million measles-related deaths have been prevented by vaccination with a measles-containing vaccine (MCV) during 2000–2023 [2] and 42 % of the world's countries have been verified for measles elimination [2,3]. However, an estimated 107,500 measles deaths still occurred in 2023 [2] mostly in children under-five [1]; children below 1 years of age have the highest risk of severe illness and death from measles compared to other age-groups [4].

**Fig. 1:**
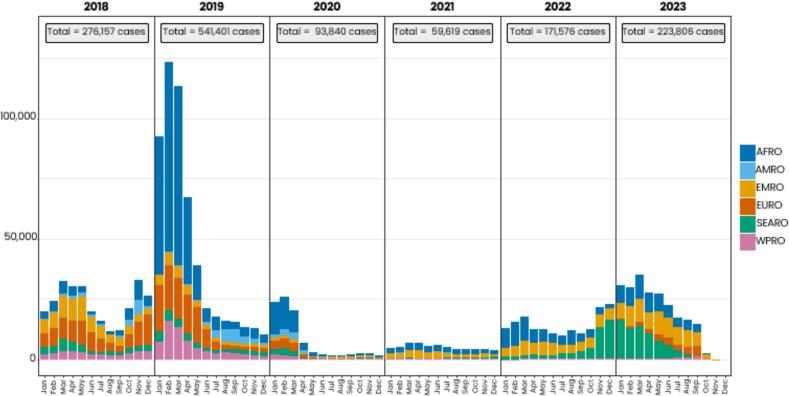
Reported measles case distribution by month and WHO region from 2018 to 2023. **Abbreviation:** AFR = African region; AMR = Americas region; EMR = Eastern Mediterranean region; EUR = European region; SEAR = Southeast Asian region; WHO=World Health Organization; WPR = Western Pacific region. **(**WHO measles reported cases and incidence).

During recent years progress has stalled: Measles cases globally have started to rise (Fig. 1), the number of countries affected by large and disruptive outbreaks has increased [5,6], and coverage with a first routinely administered MCV dose (routine MCV1) has dropped in many countries [7] in part due to the COVID-19 pandemic. These discouraging developments are slowing down progress towards measles elimination and in decreasing measles mortality among children. In addition, more women develop measles immunity through childhood vaccination rather than through measles infection and measles-vaccinated mothers may transfer lower levels of measles antibodies to their children than measles-infected mothers.

Thus, a renewed focus is needed on proven and innovative strategies to protect children against measles, especially those below 1 year of age, and on increasing coverage of routine MCV1 and a second routinely administered MCV dose (routine MCV2). The World Health Organization (WHO) recommends routine MCV1 at 9–15 months and routine MCV2 at 15–18 months of age, depending on a country's measles incidence and mortality; most LMICs recommend routine MCV1 at 9 months of age [8]. WHO's principal immunization advisory group, the Strategic Advisory Group of Experts on Immunization (SAGE), has deliberated on recommending routine MCV1 below 9 months of age (early MCV1) as a strategy to increase routine MCV1 coverage and prevent severe measles disease and death in young children [[9], [10], [11]]. However, no recommendation has so far been made to lower the age of MCV1 due to concerns about possible reduction in immunity and vaccine effectiveness and their waning. Instead, administration of a supplementary dose (MCV0) from 6 months of age is recommended in high-risk situations (e.g., measles outbreaks, refugee camps), which still needs to be followed by routine MCV1 and MCV2 at the recommended ages [8].

Since a SAGE review in 2017 [8] recent evidence has emerged on early MCV1 and at least one country, South Africa, has introduced early MCV1 at 6 months of age into their routine schedule. Thus, WHO hosted an informal technical consultation on December 6–7, 2023, in Geneva, Switzerland, with 37 attendees (24 external participants, 13 WHO staff) (supplementary material, p 1–2) to evaluate recent evidence on early MCV1 and identify evidence gaps for future policy making. Presentations covered the context, recent evidence, and modelling related to early MCV1 (supplementary material, p 3–4). Of note, potential adverse events and non-specific effects of early MCV1 [12], as well as programmatic feasibility of giving MCV1 at an earlier age, are large and independent topics. Thus, these topics were not evaluated in this meeting but would be addressed should the meeting outcome warrant revisiting WHO's current recommendation.

## 2 Context of early MCV1

### 2.1 Early MCV1 from a vaccine schedule optimization perspective

Kristen Earle from the 10.13039/100000865Bill & Melinda Gates Foundation, the United States of America, shared that many countries supported by 10.13039/100001125Gavi, the Vaccine Alliance, recommend monthly visits in a child's first year of life for non-vaccine well-child interventions, such as growth monitoring, breastfeeding promotion, and solid food introduction guidance. To ensure a high turnout at these visits, they are likely best scheduled with routine vaccination programs. Around 70 % of Gavi supported countries recommend an early routine vaccination program with visits at 6–10-14 weeks and 9 months of age. This earlier start in low-income countries (LICs) makes the schedule condensed and leaves a gap of at least 6 months between when the third dose of a pentavalent vaccine is delivered at 14 weeks of age, and when routine MCV1 is administered at 9 months of age, a period that is critical for well-child touchpoints. Furthermore, new childhood vaccines (e.g., malaria vaccine) might be challenging to fit into the existing schedule of routine vaccinations. Thus, there may be synergistic opportunities in an early MCV1 dose at 6 months of age that could enable timely visits for well-child interventions and additional contact points for new childhood vaccines.

### 2.2 Global measles surveillance data: measles cases by age-group from 2012 to 2023

**Fig. 2:**
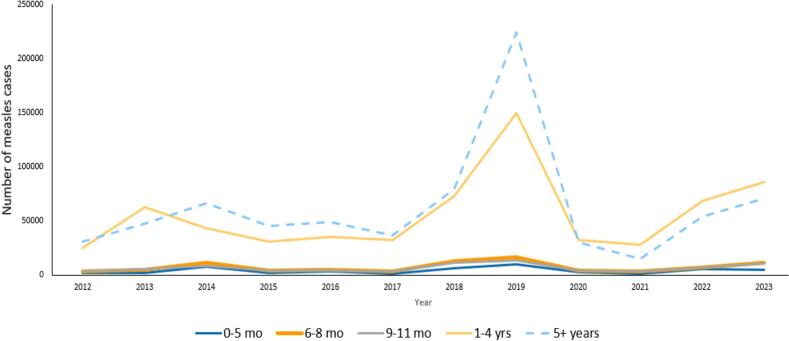
Distribution of measles cases by age-group from 2012 to 2023 based on national measles case-based surveillance data retrieved from WHO regions. The analysis included laboratory confirmed, epidemiologically linked, and clinically compatible cases. **Abbreviation:** WHO=World Health Organization. (WHO measles surveillance data).

Anna A. Minta from WHO presented the age distribution of measles cases from 2012 to 2023 based on national measles case-based surveillance data reported by WHO regions. Among countries that reported data on age during this time, the greatest number of measles cases occurred in children above 5 years of age (Fig. 2). However, the age-specific measles incidence rates were typically highest among the 6–8-month and 9–11-months old children, particularly from the African, Eastern Mediterranean, and Southeast Asian WHO regions. Furthermore, in some countries, the age-specific measles incidence rates increased during 2022–2023 among the 6–8-month and 9–11-months old, usually in conjunction with the increase in overall measles cases during 2022–2023. Measles reporting to WHO is limited by variability in reporting by WHO region and under-reporting of measles cases because of various factors (e.g., low clinical severity, cultural practices precluding seeking care from the formal healthcare sector, lack of prompt laboratory testing etc.).

In summary, the analysis of measles cases by age-group suggests that although the larger burden of cases occurs above 5 years of age, a substantial number of measles cases do occur in children below 9 months of age in endemic countries where coverage is too low to protect infants through herd immunity, and thus before WHO's current age recommendation for receiving routine MCV1.

### 2.3 Systematic review on early MCV1

Tom Woudenberg from the National Institute for Public Health and the Environment, the Netherlands, reported on two systematic reviews on early MCV1 [13,14]. The reviewed outcomes were humoral immunogenicity (including avidity), cellular immunogenicity, vaccine efficacy, vaccine effectiveness, duration of protection, safety, and immune blunting. There were generally a limited number of publications on most outcomes.

Humoral immunity data were presented as seropositivity and geometric mean concentration (GMC). The proportion of seropositive children after MCV1 at 4, 5, and 6 months of age was around 50 %, 65 %, and 75 %, respectively. Seropositivity varied by vaccine strain and presence of maternal measles antibodies. The Edmonston-Zagreb strain had the highest seropositivity followed by Connaught and Schwarz strains, respectively, and the seropositive children within the 4-months old age-group with maternal measles antibodies were less likely to seroconvert than the 4-months old without maternal measles antibodies. Children vaccinated with MCV1 at 4–8 months of age had a lower pooled geometric mean ratio (GMR) 1 month after vaccination than those vaccinated at 9 months and above (GMR 0.46, 95 % confidence interval 0.33–0.66). One randomized controlled trial (RCT) demonstrated that children who received MCV1 at 4.5 months of age had a 91 % (95 % CI 62–98 %) lower incidence of clinically diagnosed measles cases compared to children who received MCV1 later [15]. However, in a systematic review of observational studies currently being undertaken by Tom Woudenberg and colleagues (personal communication, December 6, 2024), preliminary results show a lower vaccine performance of early MCV1 with a pooled vaccine effectiveness of 58 % (95 % CI 9–80) in children who received early MCV1. No significant differences were observed in the risk of fever, rash, diarrhoea, or local reactions between children with early MCV1 versus routine MCV1.

In summary, systematic reviews on early MCV1 suggest that humoral immunity after early MCV1 is lower in younger than older children. Results of studies on whether an early MCV1 dose has lower vaccine efficacy and vaccine effectiveness are inconsistent.

### 2.4 Overview of hyporesponsiveness to early MCV and immune blunting to subsequent MCV doses

Arnaud M. Didierlaurent from the University of Geneva, Switzerland, described the mechanisms whereby impaired development of immunological memory occurs after initial active immunization with MCV. It has been observed that the total concentration of neutralizing measles antibodies and seroconversion rates may be lower when MCV is administered before 6 months versus at 9–12 months of age, even in the absence of maternal measles antibodies [16]. This is consistent with what is known about early life immune response to immunization characterized by limited early B-cell activation due to a lack of appropriate T helper cells and germinal center formation, which limits the generation of long-lived plasma cells [17]. This potential immunological development occurring between 4 and 12 months of age may determine the ability to mount an immune response at the individual level [18], but other factors than age alone, such as the child's genetic background and exposure to environmental factors or infections may also influence this development. Several studies have shown that MCV administered before 8 months of age may impact the long-term response of measles antibodies [[19], [20], [21]] which could be due to an effect of the memory B cells generated early in life, beyond the classical mechanism of immune interference by maternal antibodies, that cannot be fully recovered upon subsequent doses.

William J. Moss from the Johns Hopkins Bloomberg School of Public Health, the United States of America, gave an overview of studies of immune blunting of the second dose of measles vaccine after administration of the first dose to children younger than 9 months of age. Immune blunting, also termed immune interference, is used to refer to a poorer immune response to subsequent vaccination after prior passive or active immunization. The issue of immune blunting following subsequent MCV doses was first raised by studies in the late 1970's and early 1980's and was mentioned in several published reviews in the late 1980's and early 1990's. If true, lowering the age of MCV1 could result in lower antibody levels, lower vaccine efficacy, and more rapid waning of immunity even in children who receive a second dose of measles vaccine. However, it is important to note that MCV2 as used in the routine immunization schedule is not a booster dose but serves to immunize those children who failed to respond to MCV1.

Early studies using hemagglutinin inhibition assays showed low measles seropositivity after a second dose of measles vaccine at 15 months of age or above (49 %–60 %) when MCV1 was administered between 5 and 10 months of age, suggesting immune blunting [[22], [23], [24]]. However, subsequent studies using more sensitive assays showed high seropositivity after a second dose of measles vaccine at 15 months of age or above (94 %–98 %) when MCV1 was administered between 5 and 10 months of age, suggesting minimal or no immune blunting [[25], [26], [27]]. Minimal blunting was corroborated by recent reviews showing high seropositivity after routine MCV2 among children given early MCV1; however, these reviews also showed lower GMC of measles antibodies, lower avidity, and more rapid waning of measles antibodies after routine MCV2 in children who received a first dose of measles vaccine before 9 months of age compared to those who received MCV1 at 9 or 12 months of age [14,28,29].

In summary, the immune response to early MCV can be suboptimal, even in the absence of maternal antibodies. Evidence for immune blunting on the response to a second MCV dose after early MCV has been inconsistent, with some studies showing an equivalent immune response while others showing lower indicators of an immune response, when compared to those who received the first MCV dose at later ages (e.g., 9–12 months). Most studies were conducted over relatively short follow-up periods. Vaccine effectiveness against infection, severe disease, and death, rather than immunological indicators, is the most important measure to capture any clinical impact of potential immune blunting of early MCV.

## 3 Recent evidence on early MCV1

### 3.1 Maternally derived measles antibodies in children from birth to 6 months of age

Merryn Voysey from the University of Oxford, the United Kingdom, presented a global measles seroprevalence study characterizing the kinetics of maternal measles antibodies in young children. The study used residual serum samples from previous clinical trials and observational studies in nine countries (Mali, Gambia, Ghana, Guatemala, Pakistan, Thailand, Vietnam, the United Kingdom, the Netherlands) which enabled an assessment of measles antibodies in mothers and children at various time points (manuscript in preparation). Results from samples of up to 100 mother-child pairs per country were analysed at the National Institute for Public Health and the Environment, the Netherlands, using plaque reduction neutralization tests ***(***PRNT) assays and immunoglobulin G (IgG) titers using bead-based measles/mumps/rubella multiplex type immunoassay (MIA). The level established as the serologic correlate of protection from disease was above 0.12 IU/mL using PRNT.

**Fig. 3:**
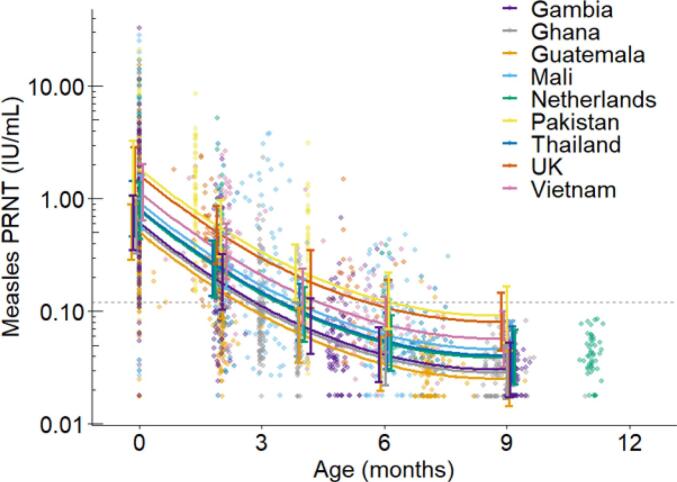
Measles antibodies in children at birth and until 12 months of age. Dashed line is the level established as the serologic correlate of protection from disease (above 0.12 IU/mL PRNT). Results were similar for MIA measurements. Data from a global seroprevalence study (2020-ongoing). **Abbreviation:** MIA = Multiplex Immunoassay; PRNT = Plaque Reduction Neutralization Test. **(**Manuscript in preparation).

**Fig. 4:**
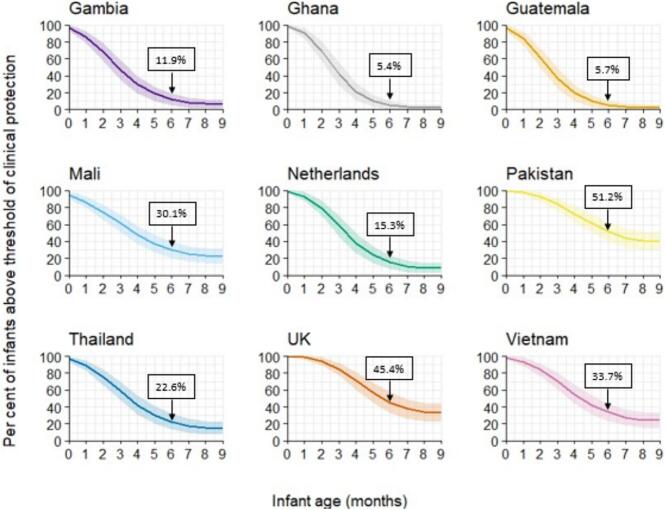
Percentage in box indicates the percentage of children at 6 months of age with measles antibodies above the threshold of 0.12 IU/mL PRNT (level established as the serologic correlate of protection from disease). Results were similar for MIA measurements. Data from a global seroprevalence study (2020-ongoing). **Abbreviation:** MIA = Multiplex Immunoassay; PRNT = Plaque Reduction Neutralization Test. (Manuscript in preparation).

Median cord or maternal transfer of measles antibodies ratios were above 1 in all countries except Pakistan, with higher transfer ratios observed in high income countries (HICs) versus LMICs. GMC of maternal measles antibodies in umbilical cord blood at birth ranged from 0.32 IU/mL (Guatemala) to 1.60 IU/mL (Pakistan) with no clear distinction between HICs versus LMICs (Fig. 3). However, by 6 months of age, and as early as 2.4 months of age (Guatemala), the level had fallen below the threshold of 0.12 IU/mL PRNT in all countries except Pakistan (Fig. 3). At 6 months of age, the percentage of children who remained above the threshold of 0.12 IU/mL PRNT ranged from 51 % in Pakistan to 5 % in Ghana (Fig. 4). Results were similar for MIA measurements. These differences across countries may relate to different maternal acquisition of measles antibodies in childhood, differences in cord-placental transfer, and the extent of vaccine-induced boosting of mothers' measles immunity. The mothers' individual vaccination status was not available; mothers participating in these studies were born during periods of routine MCV1 introduction and thus rapidly changing routine MCV1 coverage as programs embedded.

In addition, the Oxford group described an ongoing RCT in Uganda evaluating short- and long-term humoral and cellular immunogenicity where children are randomized to three schedules: MCV at 6 months and 12 months of age, MCV at 9 months and 18 months of age (routine schedule in Uganda), or MCV at 6 months and 18 months of age.

In summary, this study suggests that maternally derived measles antibodies are lost by 6 months of age in most settings.

### 3.2 Immunogenicity of early measles vaccination in the Netherlands

Rob van Binnendijk from the National Institute for Public health and the Environment, the Netherlands, presented an immunogenicity study on early vaccination with a measles-mumps-rubella-containing vaccine. In the Netherlands, routine MCV1 and MCV2 are administered at 14 months and 9 years of age, respectively. In response to a large measles outbreak in the Netherlands during 2013–2014, children aged 6–12 months from low vaccination coverage regions (below 90 %) were offered MCV0.

This measles outbreak created an opportunity to conduct an observational cohort study assessing the short- and long-term immunogenicity of MCV0 as early MCV1 (*N* = 123) in three groups: MCV at 6–8 months and 14 months of age (*n* = 46), MCV at 9–12 months and 14 months of age (*n* = 33), or MCV only at 14 months of age (*n* = 44). Blood samples were collected before MCV at 14 months of age, and more than 6 weeks, 1 year, and 3 years after MCV at 14 months of age.

**Fig. 5:**
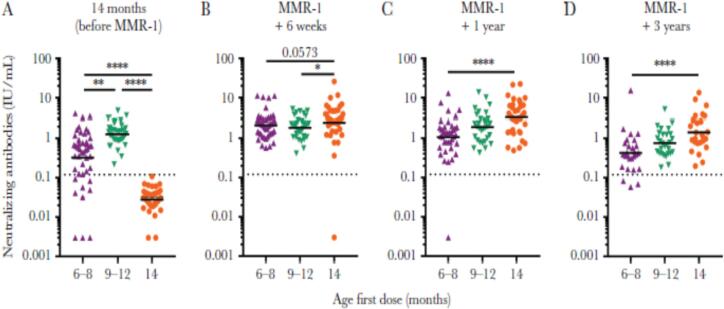
Measles antibodies in children with MCV at 6–8 months and 14 months of age, 9–12 months and 14 months of age, or only at 14 months of age, at four time points: (A) before MCV at 14 months of age, (B) 6 weeks or more after MCV at 14 months of age, (C) 1 year or more after MCV at 14 months of age, (D) 3 years or more after MCV at 14 months of age. Dashed line is the level established as the serologic correlate of protection from disease (above 0.12 IU/mL PRNT). *P ≤ .05, **P ≤ .01, and ****P ≤ .0001. Data from an observational cohort study in the Netherlands (2013-ongoing). **Abbreviation:** MMR = measles, mumps, and rubella vaccine. PRNT = Plaque Reduction Neutralization Test [21].

Results showed that 80 % of children with MCV at 6–8 months of age and 100 % of children with MCV at 9–12 months of age had measles antibodies above the protective threshold (0.12 IU/mL using PRNT) before their MCV at 14 months of age. All three groups had somewhat similar levels of measles antibodies more than 6 weeks after MCV at 14 months of age. However, more than 3 years later, the results varied; children with MCV at 6–8 months of age had the largest decrease in measles antibodies, while children with MCV only at 14 months of age, had the smallest decrease (Fig. 5) [21].

More recently derived data showed that the proportion of children at 6–7 years of age with measles antibodies above the protective threshold was lowest in the group with MCV at 6–8 months of age (30 %) and highest in the group with MCV between 9 and 12 months of age and those vaccinated only at 14 months of age (88–90 %) [30]. The authors comment that over 70 % of children vaccinated before 8·5 months lost their protective antibody levels within 6 years despite having received a repeat MCV dose at 14 months of age.

Of note, loss of measles antibodies does not equal complete loss of immune memory. Thus, clarification is needed on whether lower levels of measles antibodies correlate with a loss in cellular memory and whether this loss correlates with a greater susceptibility to measles infection and disease.

In summary, this study suggests that early MCV1 has robust humoral immunogenicity in the short-term, but the immune response wanes more quickly after MCV2 at 14 months of age in children who received an early MCV1 than children who received it at the standard age.

### 3.3 Immunogenicity of early measles vaccination in Denmark

Dorthe Maria Vittrup from Rigshospitalet, Denmark, presented an immunogenicity study on early vaccination with a measles-mumps-rubella-containing vaccine [31]. In Denmark, routine MCV1 and MCV2 are administered at 15 months and 4 years of age, respectively.

An RCT was conducted in the measles-elimination setting in Denmark from 2019 to 2021 to assess the efficacy of early MCV1 (*N* = 6500). In a subpopulation of 647 children, the immunogenicity was assessed of MCV at 6 months and 15 months of age (*n* = 290) versus MCV only at 15 months of age, (*n* = 357) using a placebo and double-blinded design. Blood samples were drawn at baseline, 3–5 weeks after MCV or placebo at 5–7 months of age, and 3–5 weeks after MCV at 15 months of age in both groups. Seropositivity rates (SPR) and GMR were measured with both enzyme-linked immunosorbent assay (ELISA) and PRNT. The level established as the serologic correlate of protection from disease was above 120 mIU/mL using PRNT and above 220 mIU/mL using ELISA IgG [32].

Shortly after MCV at 5–7 months of age, the level of measles antibodies was higher than after placebo (PRNT: GMC 120 versus 25 and SPR 47 % versus 13 %; ELISA: SPR 33 % versus 1 %) (Table 1). Shortly after MCV at 15 months of age there was no difference in the SPR between both groups (PRNT: 98 % versus 96 %; ELISA: 91 % versus 89 %). However, the GMC was about 1.5 times higher after MCV at 15 months of age among children with MCV at 5–7 months of age than after placebo (PRNT: GMC 1804 versus 1174) (Table 1). Furthermore, GMR shortly after MCV at 5–7 months of age was highest among children who might have lower levels of maternally transferred measles antibodies due to being born prematurely (gestational age below 37 weeks versus above 37 weeks: GMR 13.4 (4.9–36.1) versus 4.0 (3.2–4.9)) or from mothers with measles vaccine derived immunity (mother's birth year: after 1987 GMR 6.0 (4.3–8.4); in 1986–1987 GMR 4.6 (2.7–7.8); before 1986 GMR 2.9 (2.1–3.9)) [32]. The risk of adverse events was similar for children receiving MCV or placebo at 5–7 months of age [32].

**Table 1:** Infants with measles antibodies above the level established as the serologic correlate of protection from disease (above 120 mIU/mL using PRNT and above 220 mIU/mL using ELISA IgG). Blood samples were drawn at baseline, 3–5 weeks after MCV or placebo at 5–7 months of age, and 3–5 weeks after MCV at 15 months of age in both groups. Data from a randomized controlled trial in Denmark (2019–2021). **Abbreviation:** ELISA = Enzyme-linked immunosorbent assay; GMC = Geometric mean concentration; IgG = Immunoglobin G; MMR = Measles-mumps-rubella; PRNT = Plaque reduction neutralization test; SPR = Seropositivity rate. Adapted from [31].

	**MMR**				**Placebo**			
	Mother	Baseline	Postintervention	Postroutine	Mother	Baseline	Postintervention	Postroutine
**PRNT**								
Measles	*N* = 285	*N* = 264	N = 290	*N* = 247	*N* = 352	*N* = 327	N = 357	*N* = 316
GMC	640 (531–773)	25 (20−30)	120 (102–141)	1804 (1555–2094)	670 (581–772)	29 (25–34)	25 (22–29)	1174 (1030–1339)
SPR (%)	86	16	47	98	90	14	13	96
**ELISA IgG**								
Measles	*N* = 278	*N* = 255	*N* = 281	*N* = 256	*N* = 342	*N* = 314	*N* = 343	*N* = 328
SPR (%)	69	2	33	91	68	3	1	89

Of note, potential explanations for the higher GMR in premature children receiving early MCV1 could be that premature children have lower levels of maternally transferred measles antibodies since these are mainly transferred during the last four weeks of a full-term pregnancy. Another explanation, albeit more hypothetical, may be that the immune system of prematurely born children matures differently which influences how it responds to measles antigen exposure.

In summary, this study suggests that early MCV1 has robust humoral immunogenicity in the short-term and that short-term blunting of routine MCV2 after early MCV1 is not a concern.

### 3.4 Immunogenicity of early measles vaccination in Bangladesh

Takudzwa S. Sayi from the Centers for Disease Control and Prevention, the United States of America, presented an immunogenicity study on vaccination with a measles-rubella-containing vaccine carried out in collaboration with the International Center for Diarrhoeal Disease Research, Bangladesh, and originally conceived in response to prior studies [[33], [34], [35]] showing that children lose maternally transferred measles and rubella antibodies by 4–6 months of age. In Bangladesh, routine MCV1 and MCV2 are administered at 9 months and 15 months of age, respectively.

An RCT was conducted in 2017 with children from Bangladesh who had no history of MCV or infection with measles and rubella (manuscript in preparation). Children were enrolled at 6 months of age (*N* = 620) and randomized to receive either MCV at 6 months and 9 months of age or only at 9 months of age. Blood samples were collected from all children at 6, 9, and 11 months of age.

At baseline measles and rubella antibodies in children were similar and below the serologic correlate of protection (measles IgG ≥153mIU/mL; rubella IgG ≥9.36 IU/mL) from disease in both groups. The level of measles and rubella antibodies rose significantly after MCV at 6 months of age and was similar after MCV at 9 months of age in both groups. Seropositivity for both antigens was observed after MCV was received at 6 months of age, as well as after MCV was received at 9 months of age in both groups of children. There was no difference by study arm in the proportion of adverse events after MCV.

In summary, this study suggests that early MCV1 has robust humoral immunogenicity in the short-term, and that short-term blunting of routine MCV2 after early MCV1 is not a concern.

### 3.5 Early measles vaccination in South Africa

Daniel Kapelus from the University of Witwatersrand, South Africa, presented on the country's experience with early MCV1 implementation. In 2016, South Africa switched from routine MCV1 and MCV2 at 9 and 18 months of age to routine MCV1 and MCV2 at 6 and 12 months of age. This change was, in part, in response to a measles outbreak in 2009–11, during which 25 % of the laboratory-confirmed measles cases were detected in unvaccinated children below 9 months of age, particularly in 6–8 months old. Coinciding with the schedule change, South Africa also changed their measles vaccine from the Schwarz strain (Schwarz schedule) to a CAM-70 strain (MeasBio schedule) to allow local manufacturing.

Data from an observational cohort study were presented on the short-term immunogenicity of the early MCV1 schedule. Measles antibodies were measured before/after MCV1 (4/7 months of age) and before/after MCV2 (12/13 months of age) with the Enzygnost ELISA kit (*N* = 283). Among HIV-unexposed (HU) children, the proportion of seropositive children increased from 7 % before early MCV1 to 48 % after, increasing further to 99 % after routine MCV2 [36].

**Fig. 6:**
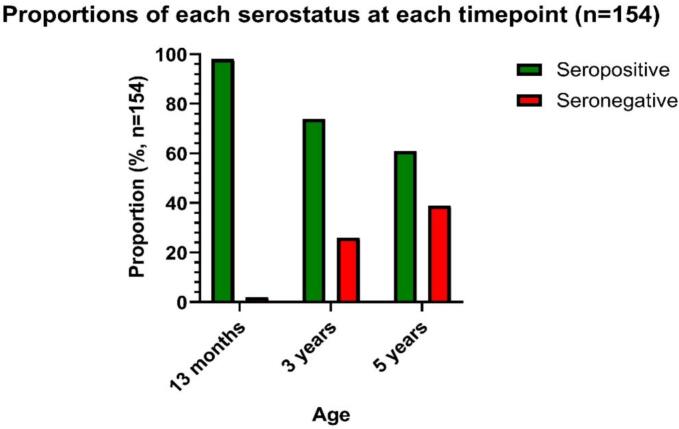
Children were vaccinated with MCV1 at 6 months of age and with MCV2 at 12 months of age. The proportion of children who were seropositive over time was measured at 13 months, 3 years and 5 years of age. The level established as the serologic correlate of protection from disease was 153 mIU/mL or above (seropositive) and < 153 mIU/mL (seronegative). Adjusted for test sensitivity differences. Data from an observational cohort study in South Africa (2005-ongoing). **Abbreviation:** MCV1 = first routinely administered dose with a measles-containing vaccine; MCV2 = second routinely administered dose with a measles-containing vaccine. (Manuscript in preparation).

Using the same cohort, data were presented on the long-term immunogenicity. Measles antibodies were measured at 3 and 5 years of age with the Euroimmun ELISA kit in children who had received early MCV1 and routine MCV2 (*n* = 154). To account for test sensitivity differences between the Enzygnost and Euroimmun ELISA kit an adjustment factor was applied (personal communication, William J. Moss, 2024). Seropositivity proportions were 74 % at 3 years of age (114/154) and 61 % at 5 years of age (94/154) with the level established as the serologic correlate of protection from disease as 153 mIU/mL or above (Fig. 6).

Using this unpublished long-term immunogenicity data on the early MCV1 schedule, a comparison was made using published [37] long-term immunogenicity data on the old schedule adjusting for test sensitivity differences; transitioning to the early MCV1 schedule may have led to lower and shorter-lived immunogenicity than the old MCV1 schedule, but potential confounding due to change from the Schwarz strain to the CAM-70 strain cannot be ruled out.

**Fig. 7:**
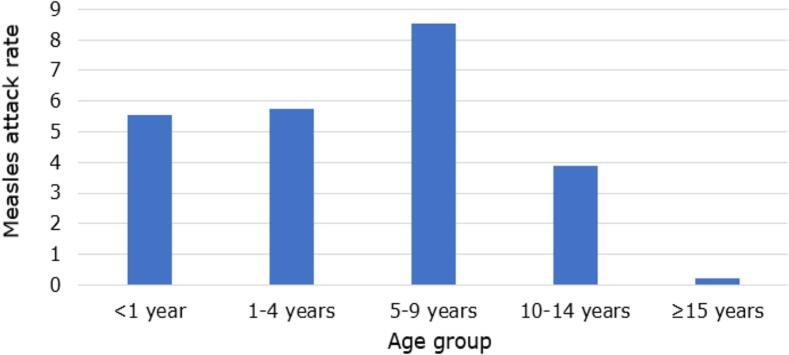
Measles incidence attack rate per 100,000 children per age group during the extended measles outbreak from week 40, 2022, to week 18, 2023, in South Africa (about five years after introducing the early MCV1 schedule). Data from an observational cohort study in South Africa (2005-ongoing). **Abbreviation:** MCV1 = first routinely administered dose with a measles-containing vaccine. (Manuscript in preparation).

Measles incidence data during a measles outbreak in 2022/23 were also shared (Fig. 7), showing that the highest attack rate was in children aged 5–9 years, all of whom could have been young enough to have received the early MCV1 schedule. However, the vaccine status of measles cases in the outbreak was not documented [38]. Moreover, the potential shift in measles cases from younger to older children could also reflect an increase in the prevalence of testing among older children rather than in disease. However, if the shift is real, it would be to shift measles cases to an older age when case-fatality rates are lower, though older infected children could be likely sources of infection for younger siblings.

In summary, this study suggests that early MCV1 has robust humoral immunogenicity in the short-term but less so in the long-term, and that children given an earlier MCV1 dose might have been at increased susceptibility of measles infection in later childhood, although the vaccination status was not documented.

### 3.6 Modelling the impact of early measles vaccination policy

Kevin McCarthy and Kurt Frey from the Bill & Melinda Gates Foundation shared an analysis of the optimal age for early MCV1 using a transmission model to balance the probability of seroconversion with the risk of measles mortality.

The model assumes that seroconversion confers lifelong protection; the seroconversion probability was modelled as a function of age and the presence or absence of maternal measles antibodies using the results of a previously published meta-analysis [13].

Measles transmission dynamics were simulated using the open-source disease modelling software EMOD [39]. These dynamics included factors such as maternally derived measles antibodies, risk of primary vaccine failure, timeliness of receiving MCV1, demographics, and coverage of MCV1 and MCV2. The optimal age for early MCV1 was modelled given an early MCV1 coverage ranging between 20 %–100 %, an expected burden of monthly measles mortality ranging between 0 and 3 cases per 100,000, and absence of routine MCV2.

**Fig. 8:**
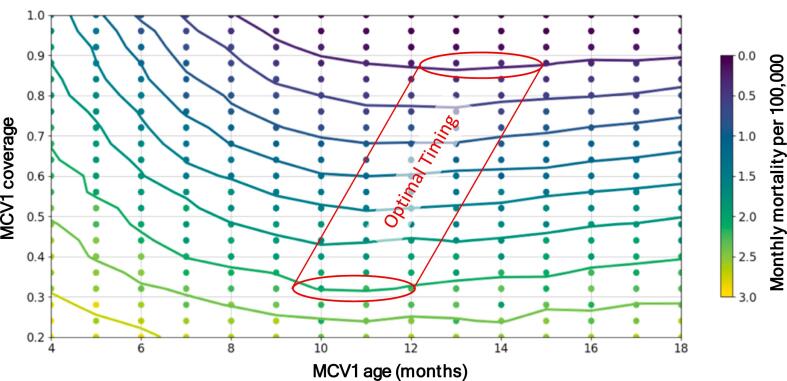
Optimal age for MCV1 in absence of MCV2 based on relation between estimated monthly measles mortality burden and MCV1 coverage. Modelling analysis using the results in [13] with modelling software in [39]. Abbreviation: MCV1 = first routinely administered dose with a measles-containing vaccine; MCV2 = second routinely administered dose with a measles-containing vaccine.

The modelling analysis showed that if early MCV1 is the only chance to be vaccinated against measles, and early MCV1 has no impact on early MCV1 coverage, then the optimal age for MCV1 is at 9 months and above at all MCV1 coverage levels (Fig. 8) because there is an increased risk of primary vaccine failure with early MCV1, resulting in an elevated lifetime risk of measles with an increased measles mortality burden.

However, if routine MCV2 is considered in the context of early MCV1 and a minimum of 75 % receive routine MCV2 at 15 months of age, then MCV1 at 6 months of age can result in a lower measles mortality burden. Furthermore, in this scenario, the window for primary vaccine failure from early MCV1 is reduced. A second dose of measles vaccination through campaigns can also mitigate the potential downsides of early MCV1, but not as effectively as routine MCV2.

Of note, future modelling analyses should account for waning of early MCV1-derived immunity, and the possible risk of a blunted response to MCV doses which would both increase the measles mortality burden. In contrast, accounting for reduced severity in breakthrough measles cases among children with primary vaccine failure after early MCV1 would decrease the measles mortality burden.

In summary, the modelling analysis suggests that MCV1 at 6 months instead of at 9 months of age would require increased MCV1 coverage to balance the loss in vaccine effectiveness, or a combination of a high risk of measles infection between 6 and 9 months of age and a very high early MCV1 and routine MCV2 coverage.

## 4 Discussion

At the WHO hosted informal technical consultation the group outlined potential pros and cons of administering early MCV1. Protecting vulnerable young children from severe measles disease and death in the short term, possibly achieving higher MCV1 coverage because coverage of all vaccines tends to be higher in younger children (although no data were available to support this for MCV), and reducing the measles burden were considered potential pros (supplementary material, p 5). However, potential cons included reduced short-term immunogenicity and effectiveness with more rapid waning, which increases the risk of a longer-term loss of population-level immunity, and immune blunting and/or imprinting after routine MCV2 (supplementary material, p 6).

Since the SAGE review of early MCV1 in 2017 [8], the age-specific measles incidence rate has increased in some countries among 6–8 months old. Furthermore, a global seroprevalence study on maternally derived measles antibodies and four immunogenicity studies on early MCV1 have been conducted in young children (supplementary material, p 7–12). This evidence suggests that children lose their maternally derived measles antibodies before 6 months of age, leaving them vulnerable to measles infection before they receive routine MCV1; although it is not clear from the data reviewed if measles is more severe during this period. The humoral immunity studies suggested a robust response shortly after early MCV1 and did not raise concern about immune blunting of routine MCV2 after early MCV1. However, the few studies that evaluated long-term humoral immunogenicity suggested that 3–7 years later, children with early MCV1 have a lower level of measles antibodies than children with standard timing of MCV1, suggesting accelerated waning. Thus, the totality of evidence on immune blunting remains inconsistent. The measles infection epidemiology from the one country that had instituted early MCV1 (concurrent with a vaccine strain change), South Africa, suggested, albeit inconclusively, that children with early MCV1 might be more susceptible to measles infection later in childhood. Moreover, the modelling analysis showed that early MCV1 would require higher coverage to balance the loss in vaccine effectiveness; however high routine MCV2 coverage can also mitigate the effect of lower immunogenicity after early MCV1. Of note, another modelling study that was published subsequent to the meeting showed that the optimal age of routine MCV1 varied depending on the epidemiological context, but was never optimal before 8 months of age, even in high transmission settings [40].

There were limitations to the evidence reviewed at the meeting. Most new studies were based on immunogenicity results and did not include effectiveness or impact of early MCV on disease incidence, either in the short or long term. Not all studies of immune blunting upon receipt of MCV2 after early MCV1 had longer term follow-up to assess potential waning. Data suggest more waning after early MCV1 over time, so follow-up studies should optimally extend to at least 5 years. The one real-world experience from South Africa of switching to early MCV1 was potentially confounded by a concomitant switch to a different measles vaccine, as well as inconsistent documentation of measles vaccination status among measles cases. Countries that choose to switch to early MCV in the future should maintain use of the same measles vaccine and carefully monitor measles vaccine status post-switch to better evaluate the response to early MCV. Furthermore, there was variability in the assays used across studies; standardizing assays to reduce variability in antibody concentration would enhance comparability.

Overall, the meeting participants thought that the recent evidence was not sufficient at this time to warrant a change to the current WHO recommendation regarding the timing of routine MCV1. However, the group expressed that as more new data emerge, the current recommendation should be revisited. More data are needed to fill evidence gaps in the following areas: disease burden (e.g., a better understanding of severe measles disease and death in children below 9 months of age), vaccine effectiveness (e.g., against severity, transmission, and over time), immunogenicity (e.g., the role of not only vaccine-induced humoral but also cellular immunogenicity), context (e.g., whether children below 9 months of age are being infected by other children in the same age group who might be protected from an early dose, or by older children who are eligible for routine MCV1 but have not been vaccinated and would therefore benefit from improved routine MCV1 coverage among children from 9 months of age), and immune blunting (e.g., immunogenicity of routine MCV2 in the short and long term after early MCV1 assessing memory B cells and plasma cells). A more extensive list of evidence gaps is provided (supplementary material, p 13–15). Aside from studies targeting these evidence gaps, it was pointed out that opportunities to study early MCV1 should be seized (e.g., through investigating transmission patterns and vaccine effectiveness during measles outbreaks and in countries choosing to introduce early MCV1). Ensuring high MCV1 and MCV2 coverage remains the priority in measles control [41].

## CRediT authorship contribution statement

**Anshu Varma:** Writing – review & editing, Writing – original draft, Conceptualization. **Shelly Bolotin:** Writing – review & editing, Writing – original draft. **Gaston De Serres:** Writing – review & editing, Writing – original draft. **Arnaud M. Didierlaurent:** Writing – review & editing, Writing – original draft. **Kristen Earle:** Writing – review & editing, Writing – original draft. **Kurt Frey:** Writing – review & editing, Writing – original draft. **Susan Hahné:** Writing – review & editing, Writing – original draft. **Daniel Kapelus:** Writing – review & editing, Writing – original draft. **L. Kendall Krause:** Writing – review & editing, Writing – original draft. **Kevin McCarthy:** Writing – review & editing, Writing – original draft. **William J. Moss:** Writing – review & editing, Writing – original draft. **Walter A. Orenstein:** Writing – review & editing, Writing – original draft. **Rob van Binnendijk:** Writing – review & editing, Writing – original draft. **Dorthe Maria Vittrup:** Writing – review & editing, Writing – original draft. **Merryn Voysey:** Writing – review & editing, Writing – original draft. **Tom Woudenberg:** Writing – review & editing, Writing – original draft. **Naor Bar-Zeev:** Writing – review & editing, Writing – original draft, Conceptualization. **Anindya S. Bose:** Writing – review & editing, Writing – original draft, Conceptualization. **Joachim Hombach:** Writing – review & editing, Writing – original draft, Conceptualization. **Mick N. Mulders:** Writing – review & editing, Writing – original draft, Conceptualization. **Laura Nic Lochlainn:** Writing – review & editing, Writing – original draft, Conceptualization. **Kezia Suwintono:** Writing – review & editing, Writing – original draft, Conceptualization. **Daniel R. Feikin:** Writing – review & editing, Writing – original draft, Conceptualization. **Natasha S. Crowcroft:** Writing – review & editing, Writing – original draft, Conceptualization.

## Funding

This meeting was funded by a grant from the Bill & Melinda Gates Foundation to the WHO (INV-005318). Disclaimer: the views, findings, and conclusions in this report are those of the authors and do not represent the positions or policies of the organizations to which the authors are affiliated.

## Declaration of competing interest

The authors declare the following financial interests/personal relationships which may be considered as potential competing interests: Authors and observers who attended the WHO meeting were subject to declaring potential competing interests. The following authors declared potential competing interests: Arnaud M. Didierlaurent is a previous employee of GlaxoSmithKline, data safety and monitoring member for AMC Biologicals, consultant for Sanofi, and speaker for Sanofi and Merck. He is a member of the Emergency Use Listing WHO Technical Advisory Group. His current research unit has research collaboration agreements with Moderna, Sanofi, and GSK; Dorthe Maria Vittrup receives financial compensation for academic teaching and public speaking on measles; L. Kendall Krause, Kevin McCarthy, Kristen Earle, and Kurt Frey are employed by the Bill & Melinda Gates Foundation which funded the meeting; Shelly Bolotin is employed by public sector entities with an interest in vaccine research and surveillance. She serves as the Director of the Centre for Vaccine Preventable Diseases at the University of Toronto. The center has received donations from Merck, Pfizer and Sanofi and adheres to governance processes at the University of Toronto to ensure it operates independently. As the Director, she has advocated for increasing immunization coverage and has publicly made statements about the safety and effectiveness of specific vaccines, including MCV. She has been funded by various Canadian Federal and other initiatives; Walter A. Orenstein is a consultant for Sanofi. The following authors declared no potential competing interests: Daniel Kapelus; Gaston De Serres; Merryn Voysey; Rob van Binnendijk; Susan Hahné; Tom Woudenberg; William J. Moss. The following observers declared potential competing interests: Ana Leticia Nery is employed by the 10.13039/100000865Bill & Melinda Gates Foundation which funded this meeting; Anthony Scott receives research support for measles-related work from the 10.13039/501100000265Medical Research Council of the United Kingdom and 10.13039/100000865Bill & Melinda Gates Foundation; Gerald Bright Businge receives research support for measles-related work from the 10.13039/100000865Bill & Melinda Gates Foundation. The following observers declared no potential competing interests: Lien Anh Ha Do; Joy Lee; Katrina Kretsinger.

## Data Availability

Data availability: Not relevant

## Appendix A Supplementary data

Supplementary material: **1-2:Supplementary table 1:** Information on participants in the World Health Organization informal technical consultation on December 6-7, 2023, in Geneva, Switzerland - **Caption for supplementary appendix page 3-4:Supplementary table 2:** Meeting agenda for the World Health Organization informal technical consultation on December 6-7, 2023, in Geneva, Switzerland - **Caption for supplementary appendix page 5:Supplementary table 3:** Outline of potential pros of administering early MCV1 - **Caption for supplementary appendix page 6:Supplementary table 4:** Outline of potential cons of administering early MCV1 - **Caption for supplementary appendix page 7-12:Supplementary table 5:** Overview of new evidence. Results from a global measles seroprevalence study and four humoral immunogenicity studies of early measles vaccination - **Caption for supplementary appendix page 13-15:Supplementary table 6:** Overview of evidence gaps on early MCV1. Image 1

## References

[1] (2024). World Health Organization. Measles [Internet]. https://www.who.int/news-room/fact-sheets/detail/measles.

[2] Minta A.A., Ferrari M., Antoni S., Portnoy A., Sbarra A., Lambert B. (2024). Progress toward measles elimination — worldwide, 2000–2023. MMWR Morb Mortal Wkly Rep.

[3] WHO (2023).

[4] Sbarra A.N., Mosser J.F., Jit M., Ferrari M., Ramshaw R.E., O’Connor P. (2023). Estimating national-level measles case–fatality ratios in low-income and middle-income countries: an updated systematic review and modelling study. Lancet Glob Health.

[5] WHO (2023).

[6] Centers for Disease Control and Prevention. Immunity Profiles [Internet] 2023. Available from: https://www.mdpi.com/2076-393X/12/8/937.

[7] (2023). WHO, UNICEF. WHO UNICEF Immunization Coverage Estimates 2022 revision [Internet]. https://cdn.who.int/media/docs/default-source/immunization/immunization-coverage/wuenic_notes.pdf?sfvrsn=88ff590d_16&download=true.

[8] World Health Organization (2019). Measles vaccines: WHO position paper, April 2017 - recommendations. Vaccine.

[9] (2025). Meeting of the strategic advisory Group of Experts on immunization, November 2013: conclusions and recommendations [Internet]. https://www.who.int/publications/i/item/WER8901.

[10] (2024). Meeting of the Strategic Advisory Group of Experts on Immunization, October 2015: conclusions and recommendations [Internet]. https://www.who.int/publications/i/item/WER9050.

[11] (2024). Meeting of the Strategic Advisory Group of Experts on Immunization, October 2016: conclusions and recommendations [Internet]. https://www.who.int/publications/i/item/WER9148.

[12] Benn C.S., Fisker A.B., Rieckmann A., Sørup S., Aaby P. (2020). Vaccinology: time to change the paradigm?. Lancet Infect Dis.

[13] Nic Lochlainn L.M., de Gier B., van der Maas N., Strebel P.M., Goodman T., van Binnendijk R.S. (2019). Immunogenicity, effectiveness, and safety of measles vaccination in infants younger than 9 months: a systematic review and meta-analysis. Lancet Infect Dis.

[14] Nic Lochlainn L.M., de Gier B., van der Maas N., van Binnendijk R., Strebel P.M., Goodman T. (2019). Effect of measles vaccination in infants younger than 9 months on the immune response to subsequent measles vaccine doses: a systematic review and meta-analysis. Lancet Infect Dis.

[15] Martins C.L., Garly M.L., Balé C., Rodrigues A., Ravn H., Whittle H.C. (2008). Protective efficacy of standard Edmonston-Zagreb measles vaccination in infants aged 4.5 months: interim analysis of a randomised clinical trial. BMJ.

[16] Gans H.A., Yasukawa L.L., Alderson A., Rinki M., DeHovitz R., Beeler J. (2004). Humoral and cell-mediated immune responses to an early 2-dose measles vaccination regimen in the United States. J Infect Dis.

[17] Mohr E., Siegrist C.A. (2016). Vaccination in early life: standing up to the challenges. Curr Opin Immunol.

[18] Pieren D.K.J., Boer M.C., de Wit J. (2022). The adaptive immune system in early life: the shift makes it count. Front Immunol.

[19] Gans H.A., Yasukawa L.L., Sung P., Sullivan B., DeHovitz R., Audet S. (2013). Measles humoral and cell-mediated immunity in children aged 5–10 years after primary measles immunization administered at 6 or 9 months of age. J Infect Dis.

[20] Njie-Jobe J., Nyamweya S., Miles D.J.C., van der Sande M., Zaman S., Touray E. (2012). Immunological impact of an additional early measles vaccine in Gambian children: responses to a boost at 3 years. Vaccine.

[21] Brinkman I.D., De Wit J., Smits G.P., Ten Hulscher H.I., Jongerius M.C., Abreu T.C. (2019). Early measles vaccination during an outbreak in the Netherlands: short-term and long-term decreases in antibody responses among children vaccinated before 12 months of age. J Infect Dis.

[22] Wilkins J., Wehrle P.F. (1979). Additional evidence against measles vaccine administration to infants less than 12 months of age: altered immune response following active/passive immunization. J Pediatr.

[23] Linnemann C.C.J., Dine M.S., Roselle G.A., Askey P.A. (1982). Measles immunity after revaccination: results in children vaccinated before 10 months of age. J Pediatr.

[24] Black F.L., Berman L.L., Libel M., Reichelt C.A., Pinheiro F.D., Travassos da Rosa A. (1984). Inadequate immunity to measles in children vaccinated at an early age: effect of revaccination. Bull World Health Organ.

[25] Murphy M.D., Brunell P.A., Lievens A.W., Shehab Z.M. (1984). Effect of early immunization on antibody response to reimmunization with measles vaccine as demonstrated by enzyme-linked immunosorbent assay (ELISA). J Pediatr.

[26] McGraw T.T. (1986). Reimmunization following early immunization with measles vaccine: a prospective study. J Pediatr.

[27] Stetler H.C., Orenstein W.A., Bernier R.H., Herrmann K.L., Sirotkin B., Hopfensperger D. (1986). Impact of revaccinating children who initially received measles vaccine before 10 months of age. J Pediatr.

[28] Carazo S., Billard M.N., Boutin A., De Serres G. (2020). Effect of age at vaccination on the measles vaccine effectiveness and immunogenicity: systematic review and meta-analysis. BMC Infect Dis.

[29] Xu J., Doyon-Plourde P., Tunis M., Quach C. (2021). Effect of early measles vaccination on long-term protection: a systematic review. Vaccine.

[30] van der Staak M., ten Hulscher H.I., Nicolaie A.M., Smits G.P., de Swart R.L., de Wit J. (2025). Long-term Dynamics of Measles Virus–Specific Neutralizing Antibodies in Children Vaccinated Before 12 Months of Age. Clin Infect Dis.

[31] Vittrup D.M., Laursen A.C.L., Malon M., Soerensen J.K., Hjort J., Buus S. (2020). Measles-mumps-rubella vaccine at 6 months of age, immunology, and childhood morbidity in a high-income setting: study protocol for a randomized controlled trial. Trials.

[32] Vittrup D.M., Jensen A., Sørensen J.K., Zimakoff A.C., Malon M., Charabi S. (2024). Immunogenicity and reactogenicity following MMR vaccination in 5–7-month-old infants: a double-blind placebo-controlled randomized clinical trial in 6540 Danish infants. eClinicalMedicine.

[33] Gagneur A., Pinquier D. (2010). Early waning of maternal measles antibodies: why immunization programs should be adapted over time. Expert Rev Anti-Infect Ther.

[34] Leuridan E., Van Damme P. (2007). Passive transmission and persistence of naturally acquired or vaccine-induced maternal antibodies against measles in newborns. Vaccine.

[35] Leuridan E., Hens N., Hutse V., Ieven M., Aerts M., Van Damme P. (2010). Early waning of maternal measles antibodies in era of measles elimination: longitudinal study. BMJ.

[36] Mutsaerts E.A.M.L., Nunes M.C., Bhikha S., Ikulinda B.T., Boyce W., Jose L. (2019). Immunogenicity and safety of an early measles vaccination schedule at 6 and 12 months of age in human immunodeficiency virus (HIV)–unexposed and HIV-exposed, uninfected south African children. J Infect Dis.

[37] Mutsaerts E.A.M.L., Nunes M.C., Van Rijswijk M.N., Klipstein-Grobusch K., Otwombe K., Cotton M.F. (2019). Measles immunity at 4.5 years of age following vaccination at 9 and 15–18 months of age among human immunodeficiency virus (HIV)–infected, HIV-exposed–uninfected, and HIV-unexposed children. J Clin Infect Dis.

[38] National Institute for Communicable Diseases (2023). Interim Situation Report, 12 May 2023 [Internet]. https://www.nicd.ac.za/wp-content/uploads/2023/05/South-African-measles-outbreak-12-May-2023.pdf.

[39] Institute for Disease Modeling (2025). Modeling Software EMOD V2.22 [Internet]. https://github.com/InstituteforDiseaseModeling/EMOD.

[40] Goult E., Barrero Guevara L.A., Briga M. (2024). Domenech de Cellès M. Estimating the optimal age for infant measles vaccination. Nat Commun.

[41] Yitbarek K., Mahimbo A., Bobo F.T., Woldie M., Sheel M., Frawley J. (2025). Impact of measles vaccination strategies on vaccination rates in low-income and middle-income countries: a systematic review and meta-analysis. BMJ Glob Health.

