# Supplementary material for: What is the current evidence base for measles vaccination earlier than 9 months of age?: Report from an informal technical consultation of the World Health Organization
Source: Vaccine. 2025 May 31;57:None. doi: 10.1016/j.vaccine.2025.127187 (PMC12176663; doi:10.1016/j.vaccine.2025.127187)
Supplement: Supplementary file 1 — Supplementary material: 1-2:Supplementary table 1: Information on participants in the World Health Organization informal technical consultation on December 6-7, 2023, in Geneva, Switzerland - Caption for supplementary appendix page 3-4:Supplementary table 2: Meeting agenda for the World Health Organization informal technical consultation on December 6-7, 2023, in Geneva, Switzerland - Caption for supplementary appendix page 5:Supplementary table 3: Outline of potential pros of administering early MCV1 - Caption for supplementary appendix page 6:Supplementary table 4: Outline of potential cons of administering early MCV1 - Caption for supplementary appendix page 7-12:Supplementary table 5: Overview of new evidence. Results from a global measles seroprevalence study and four humoral immunogenicity studies of early measles vaccination - Caption for supplementary appendix page 13-15:Supplementary table 6: Overview of evidence gaps on early MCV1. [file mmc1.docx]

**Meeting attendees and roles**

| **External members - in-person attendance** | |
| --- | --- |
| Shelly Bolotin | University of Toronto, Canada |
| Arnaud M. Didierlaurent | University of Geneva, Switzerland |
| Kristen Earle | Bill & Melinda Gates Foundation, the United States of America |
| Daniel Kapelus | University of Witwatersrand, South Africa |
| L. Kendall Krause | Bill & Melinda Gates Foundation, the United States of America |
| William J. Moss | Johns Hopkins Bloomberg School of Public Health, the United States of America |
| Walter A. Orenstein | Emory University, the United States of America |
| Paul A. Rota | Centers for Disease Control and Prevention, the United States of America |
| Takudzwa S. Sayi | Centers for Disease Control and Prevention, the United States of America |
| Rob van Binnendijk | National Institute for Public Health and the Environment, the Netherlands |
| Dorthe Maria Vittrup | Rigshospitalet, Denmark |
| Merryn Voysey | University of Oxford, the United Kingdom |
| Tom Woudenberg | National Institute for Public Health and the Environment, the Netherlands |
| **External members - virtual attendance** | |
| Gaston De Serres | Laval University, Canada |
| Kurt Frey | Bill & Melinda Gates Foundation, the United States of America |
| Susan Hahné | National Institute for Public Health and the Environment, the Netherlands |
| Kevin McCarthy | Bill & Melinda Gates Foundation, the United States of America |
| Katherine Rosenfeld | Bill & Melinda Gates Foundation, the United States of America |
| **External observers - virtual attendance** | |
| Gerald Bright Businge | Makerere University-Johns Hopkins Research Collaboration, Uganda |
| Lien Anh Ha Do | Murdoch Children's Research Institute, Australia |
| Katrina Kretsinger | Self-employed Public Health consultant, the United States of America |
| Joy Lee | Bill & Melinda Gates Foundation, the United States of America |
| Ana Leticia Nery | Bill & Melinda Gates Foundation, the United States of America |
| Anthony Scott | London School of Hygiene & Tropical Medicine, the United Kingdom |
| **World Health Organization members - in-person or virtual attendance** | |
| Anindya S. Bose | World Health Organization |
| Natasha S. Crowcroft | World Health Organization |
| Daniel R. Feikin | World Health Organization |
| Anna A. Minta | World Health Organization |
| Mick N. Mulders | World Health Organization |
| Laura Nic Lochlainn | World Health Organization |
| Patrick M. O'Connor | World Health Organization |
| Kezia Suwintono | World Health Organization |
| Anshu Varma | World Health Organization |
| **World Health Organization observers - virtual attendance** | |
| Roodly W. Archer | World Health Organization |
| Syeda Kanwal Aslam | World Health Organization |
| Sudhir Khanal | World Health Organization |
| Judith van Holten | World Health Organization |

**Meeting agenda for Informal Technical Consultation on Early Measles Vaccination**

| **DECEMBER 6 - DAY 1** | |  |  |
| --- | --- | --- | --- |
| **Session 1 - Welcome** | |  |  |
| 09:30-09:50 | World Health Organization - opening | Natasha S. Crowcroft | World Health Organization, Switzerland |
| 09:50-10:00 | Chair - opening | Walter A. Orenstein | Emory University, the United States of America |
| **Session 2 - Background and Epidemiology** | |  |  |
| 10:00-10:10 | Early MCV1 in the context of optimized scheduling | Kristen Earle | Bill & Melinda Gates Foundation, the United States of America |
| 10:10-10:40 | Global measles surveillance review: measles cases among infants, 2013-2023 | Anna A. Minta | World Health Organization |
| *10:40-11.00* | *Break (tea & coffee)* |  |  |
| 11:00-11:30 | Systematic literature review and meta-analyses of measles vaccination among infants aged 6 months and below | Tom Woudenberg | National Institute for Public Health and the Environment, the Netherlands |
| 11:30-12:00 | Q & A | Walter A. Orenstein |  |
| *12:00-13:00* | *Lunch* |  |  |
| **Session 3 - Immunogenicity studies** | |  |  |
| 13:00-13:20 | Dutch study on Early MCV1 | Rob van Binnendijk | National Institute for Public Health and the Environment, the Netherlands |
| 13:20-13:30 | Q & A | Walter A. Orenstein |  |
| *13:30-13:40* | *Break (stretch)* |  |  |
| 13:40-14:00 | Danish RCT on Early MMR | Dorthe Maria Vittrup | Rigshospitalet, Denmark |
| 14:00-14:10 | Q & A | Walter A. Orenstein |  |
| *14:10-14:40* | *Break (tea & coffee)* |  |  |
| 14:40-15:00 | Earlier prime-boost measles administration in Uganda (The BoostMe Study) | Merryn Voysey | University of Oxford, the United Kingdom |
| 15:00-15:10 | Q & A | Walter A. Orenstein |  |
| *15:10-15:20* | *Break (stretch)* |  |  |
| 15:20-15:40 | Assessing immunogenicity and safety of MR vaccine at 6 months & 9 months: A randomized, open-label, clinical trial in Bangladesh | Takudzwa S. Sayi | Centers for Disease Control and Prevention, the United States of America |
| 15:40-15:50 | Q & A | Walter A. Orenstein |  |
| 15:50-16:05 | High-level recap of the day | Walter A. Orenstein |  |
| 16:05-16:15 | Closing remarks | Walter A. Orenstein & Natasha S. Crowcroft |  |
| *19:00* | *Dinner* |  |  |
| **DECEMBER 7 - DAY 2** | |  |  |
| **Session 4 - Immunological blunting** | |  |  |
| 08:30:08:40 | Summary on immune blunting | William J. Moss | Johns Hopkins Bloomberg School of Public Health, the United States of America |
| 08:40-08:50 | Immunological considerations and research gaps | Arnaud M. Didierlaurent | University of Geneva, Switzerland |
| 08:50-09:00 | Q & A | Walter A. Orenstein |  |
| **Session 5 - Country experience** | |  |  |
| 09:00-10:00 | Early MCV1 in South Africa | Daniel Kapelus | University of Witwatersrand, South Africa |
| 10:00-10:20 | Q & A | Walter A. Orenstein |  |
| *10:20-10:40* | *Break (tea & coffee)* |  |  |
| **Session 6 - Modelling** | |  |  |
| 10:40-11:40 | Key parameters and tradeoffs in early MCV1 modeling | Kevin McCarthy & Kurt Frey | Bill & Melinda Gates Foundation, the United States of America |
| 11:40-12:00 | Q & A | Walter A. Orenstein |  |
| *12:00-13:00* | *Lunch* |  |  |
| **Session 7 - Discussion of available evidence and gaps** | |  |  |
| 13:00-15:00 | Knowledge gaps | All participants |  |
|  | Pros and cons of changing to early MCV1 | All participants |  |
|  | Conclusions based on available evidence | All participants |  |
| *15:00-15:30* | *Break (tea & coffee)* |  |  |
| 15:30-15:45 | High-level recap of the day | Walter A. Orenstein |  |
| 15:45-16:15 | Closing | Walter A. Orenstein & Natasha S. Crowcroft |  |

**Potential pros of early MCV1**

| **Potential pros**^a^ | **Brief explanation** |
| --- | --- |
| Protecting young children from measles infection, severe disease, and death, in the short-term | Younger children are generally vulnerable and susceptible to severe measles disease and death aside from complications |
| Obtaining a higher measles vaccination coverage | Children at 6 months are easier to reach than at 9 months of age. Furthermore, a 6-month well-child visit may allow increased attendance |
| Reducing measles burden | Burden of measles severe disease and death and complications in the short- and long-term is reduced |
| **Abbreviation:** MCV=measles-containing vaccine | |
| ^a^Pros were identified through an online survey during the plenary session of day 2. Seventy percent of the attendees responded (26 out of 37). Subsequently, the organizing committee thematized the pros. Each theme was ranked according to the number of times a theme could be extracted from an attendee’s response. | |

**Potential cons of early MCV1**

| **Potential cons**^a^ | **Brief explanation** |
| --- | --- |
| Reduced short-term immunogenicity and vaccine effectiveness with more rapid waning | If immunity and vaccine effectiveness are not sustained even after routine MCV2/MCV3/MCV4 will be warranted to respond to a new immunity gap since susceptibility will shift to a later age. Furthermore, there is a risk of a longer-term loss of population immunity. This will translate into an increased measles burden and bring about global vaccine supply and financial issues |
| Blunting and/or imprinting after routine MCV2 | Blunting of the immune response may translate into a significant clinical problem. Imprinting can result in lower levels of measles antibodies and accelerate risk of vaccine failure, especially given low coverage |
| **Abbreviation:** MCV=measles-containing vaccine | |
| ^a^Cons were identified through an online survey during the plenary session of day 2. Seventy percent of the attendees responded (26 out of 37). Subsequently, the organizing committee thematized the cons. Each theme was ranked according to the number of times a theme could be extracted from an attendee’s response. | |

**Overview of new evidence. Results from a global measles seroprevalence study and four humoral immunogenicity studies of early measles vaccination**

| **Country** | **Design** | **Intervention** | **Comparator** | **Short-term result** | **Long-term result** | **Plans** |
| --- | --- | --- | --- | --- | --- | --- |
| Mali, Gambia, Ghana, Guatemala, Pakistan, Thailand, Vietnam, the United Kingdom, the Netherlands | Seroprevalence study from 2020- | NR | NR | Median cord/maternal transfer of measles antibodies ratios were above 1 in all countries except Pakistan, with higher transfer ratios observed in HICs versus LMICs. GMC of maternal measles antibodies in umbilical cord blood at birth ranged from 0.32 IU/mL (Guatemala) to 1.60 IU/mL (Pakistan) with no clear distinction between HICs versus LMICs (figure 3) | However, by 6 months of age, and as early as 2.4 months of age (Guatemala), the level had fallen below the threshold of 0.12 IU/mL PRNT in all countries except Pakistan (figure 3). At 6 months of age, the percentage of children who remained above the threshold of 0.12 IU/mL PRNT ranged from 51% in Pakistan to 5% in Ghana (figure 4) | Manuscript in preparation |

| **Country** | **Design** | **Intervention** | **Comparator** | **Short-term result** | **Long-term result** | **Plans** |
| --- | --- | --- | --- | --- | --- | --- |
| The Netherlands | Observational cohort study, humoral immunogenicity from 2013-ongoing  The level established as the serologic correlate of protection from disease was above 0.12 IU/mL using PRNT. | MCV at 6-8 months and 14 months of age | versus at 9-12 months and 14 months of age, or only at 14 months of age | Results showed that 80% of children with MCV at 6-8 months of age and 100% of children with MCV at 9-12 months of age had measles antibodies above the threshold before their MCV at 14 months of age. All three groups had somewhat similar levels of measles antibodies more than 6 weeks after MCV at 14 months of age. | However, more than 3 years later, the results varied; children with MCV at 6-8 months of age had the largest decrease in measles antibodies, while children with MCV only at 14 months of age, had the smallest decrease (figure 5). More recently derived data showed that the proportion of children at 6-7 years of age with measles antibodies above the protective threshold was lowest in the group with MCV at 6-8 months of age (30%) and highest in the group with MCV between 9-12 months of age and those vaccinated only at 14 months of age (88-90%). | Ethical approval to retrieve follow-up data on cohort until 11 years of age is being sought |

| **Country** | **Design** | **Intervention** | **Comparator** | **Short-term result** | **Long-term result** | **Plans** |
| --- | --- | --- | --- | --- | --- | --- |
| Denmark | RCT, humoral immunogenicity from 2019-2021  The level established as the serologic correlate of protection from disease was above 120 mIU/mL using PRNT and above 220 mIU/mL using ELISA IgG | MCV at 5-7 months and 15-months of age | versus only at 15 months of age versus placebo | Shortly after MCV at 5-7 months of age, the level of measles antibodies was higher than after placebo (PRNT: GMC 120 versus 25 and SPR 47% versus 13%; ELISA: SPR 33% versus 1%) (table 1). Shortly after MCV at 15 months of age there was no difference in the SPR between both groups (PRNT: 98% versus 96%; ELISA: 91% versus 89%). However, the GMC was about 1.5 times higher after MCV at 15 months of age among children with MCV at 5-7 months of age than after placebo (PRNT: GMC 1804 versus 1174) (table 1) | NA | Short-term cellular immunogenicity also studied but results not available, yet. Funding being sought to measure long-term humoral and cellular immunogenicity |
| **Country** | **Design** | **Intervention** | **Comparator** | **Short-term result** | **Long-term result** | **Plans** |
| Bangladesh | RCT, humoral  immunogenicity from 2017-ongoing  The level established as the serologic correlate of protection from disease was for measles IgG ≥153mIU/mL and for rubella IgG 9.36IU/mL) | MCV at 6 months and 9 months of age | versus only at 9 months of age | At baseline measles and rubella antibodies in children were similar and below the level established as the serologic correlate of protection from disease in both groups. The level of measles and rubella antibodies rose significantly after MCV at 6 months of age and was similar after MCV at 9 months of age in both groups. Most children were seropositive for both antigens after MCV at 6 months of age, also after MCV at 9 months of age, in both groups. | NA | Manuscript in preparation.  Long term follow up of both groups is not possible in Bangladesh |

| **Country** | **Design** | **Intervention** | **Comparator** | **Short-term result** | **Long-term result** | **Plans** |
| --- | --- | --- | --- | --- | --- | --- |
| South Africa | Observational cohort study, humoral immunogenicity 2005-ongoing | MCV at 6 months and 12 months of age | NR | Among HIV-unexposed (HU) children, the proportion of seropositive children increased from 7% before early MCV1 to 48% after, increasing further to 99% after routine MCV2 (35). | Seropositivity proportions were 74% at 3 years of age (114/154) and 61% at 5 years of age (94/154) with the level established as the serologic correlate of protection from disease as 153 mIU/mL or above (figure 6).  Transitioning to the early MCV1 schedule may have led to lower and shorter-lived immunogenicity than the old MCV1 schedule.  The highest measles attack rate was in children aged 5–9 years, all of whom could have been young enough to have received the early MCV1 schedule (37). | Manuscript in preparation.  Conduct vaccine effectiveness analyses and continue follow-up in children who received the early schedule (~7 years old now) allowing assessment of MCV booster impact |

| **Country** | **Design** | **Intervention** | **Comparator** | **Short-term result** | **Long-term result** | **Plans** |  |
| --- | --- | --- | --- | --- | --- | --- | --- |
| Uganda | RCT, humoral and cellular immunogenicity 2023-ongoing | MCV at 6 months and 12 months of age | versus at 6 months and 18 months versus at 9 months and 18 months (routine schedule in Uganda) of age | NA | NA | First RCT to assess the immunogenicity of MCV1 at 6 months versus 9 months of age, followed by routine MCV2 in the second year of life.  Recruitment started in November 2023 and completion expected by mid-2024 |  |
| **Abbreviations:** GMC=Geometric mean concentration; HICs=High-income countries; HIV=Human immunodeficiency virus; LMICs=Low- and middle-income countries; MCV=measles containing vaccine; NA=Not available; NR=Not relevant; PRNT=Plaque reduction neutralization tests; RCT=randomized controlled trial; SPR=seroprotection | | | | | | |  |
|  |  |  |  |  |  |  |  |

**Overview of evidence gaps on early MCV1**

| **Gaps^a^** | **Potential research topics** |
| --- | --- |
| Disease burden | Prevalence and incidence of measles infection and severe measles disease and death according to vaccination status and risk setting (low vaccination coverage, malnutrition, limited healthcare access) in young children |
|  | Prevalence and incidence of measles infection and severe measles disease and death in primary and secondary vaccine failures after early MCV1 versus routine MCV1 |
|  | Prevalence and incidence of breakthrough infections and their severity after early MCV1 versus routine MCV1 |
|  | Clarify if waning cellular and humoral immunity translates into an increased and severe measles disease and death burden at the individual and community level |
| Vaccine effectiveness | Overall and by age |
|  | MCV1 at 6 months versus 9 months versus more than 12 months of age in the short- and long-term |
|  | Routine MCV2 after early MCV1 at 6 months versus 9 months versus more than 12 months of age in the short- and long-term |
|  | Early MCV1 in a 3 versus 2-dose schedule in the short- and long-term |
|  | Early MCV1 against severity, against transmission, in high versus low-risk populations, by elimination and endemicity status, by outbreak frequency, by vaccine strain, by risk and burden of malaria and HIV |
|  | Modelling waning vaccine effectiveness after early MCV1 |
| Immunogenicity | MCV1 at 6 months versus 9 months versus more than 12 months of age in the short- and long-term (e.g., at least 5 years) |
|  | Routine MCV2 after early MCV1 at 6 months versus 9 months versus more than 12 months of age in the short- and long-term (e.g., at least 5 years) |
|  | Early MCV1 in a 3 versus 2-dose schedule in the short- and long-term (e.g., at least 5 years) |
|  | Difference between early MCV1 induced versus early measles infection induced immunity |
|  | Routine MCV2 response to any immune deficit after early MCV1 |
|  | Seronegative after early MCV1 and immune memory level |
|  | Immune profile of early MCV1 low responders across age and settings using baseline markers and conducting in-depth analyses of the innate and adaptive response |
|  | Cellular and humoral immune response and immunological memory after early MCV1 by age, population, nutritional status, country income group, vaccine strain, breakthrough infection and severity, elimination and endemicity status, outbreak frequency, burden of malaria and HIV - in the short- and long-term |
|  | Compensatory role of cellular immunity given waning humoral immunity after early MCV1 |
|  | Relation between early measles infection and immunity maturation rate |
|  | Translation of early MCV1 into further reduced transfer of maternally derived measles antibodies to generations born in coming decades |
|  | Modelling waning immune response after early MCV1 |
| Context | Transmission patterns of measles infection. Whether children below 9 months of age are being measles infected by other children in the same age group (who might even be protected from an early dose) or by older children who are eligible for measles vaccination but have not been vaccinated and would therefore benefit from improved routine MCV1 coverage among children +9 months of age |
|  | Transmission patterns of breakthrough infections after early MCV1 versus routine MCV1 |
|  | Infectiousness of breakthrough infections after early MCV1 versus routine MCV1 |
|  | Coverage of routine MCV1 and MCV2 in countries where early MCV1 is considered; do both or any of the coverages increase or decrease after early MCV1 |
|  | Proportion of on-time routine MCV1 and MCV2 in current schedule |
| Evaluation methodology | Studies in settings similar to South Africa where early MCV1 is implemented, and surveillance data are of high quality and in outbreaks to investigate transmission patterns and vaccine effectiveness |
|  | For modelling, use quantitative assay measures instead of dichotomization of data into e.g., presence or absence of maternally derived measles antibodies |
|  | Make the definition and measurement of immune blunting more precise. E.g. 1) a lower response at a lower age due to interference of maternally derived measles antibodies and/or skewed immunity 2) Imprinting associated with declining immunity persisting upon subsequent doses by status of maternal measles antibodies (assess difference in B-cell quality between early and routine MCV1 measuring plasma blasts, measles antibodies, somatic hypermutation, and clonal diversity) 3) Class switching 4) General difference between immune blunting and immune imprinting and correlation with vaccine failure 5) Standardizing laboratory methods and assays to reduce variability in antibody concentration and percentages above protective thresholds |
|  | Prediction models for setting specific long-term vaccine effectiveness and immune response after early MCV1 |
|  | For systematic review purposes, it would be helpful if individual studies do not report at an aggregated level to allow inference based on age of vaccination |
|  | Prolong follow-up periods for any outcomes since these are generally very short (e.g. at least 5 years) |
|  | Improve ways to retrieve data on the history of maternal measles infection and/or vaccination |
|  | Functionality of measles-specific memory B cell pool (boostability, clonality, amount of somatic hyper mutations) generated by early MCV1 and a better characterization of the impact of early MCV1 on measles-specific T cell response, in particular T follicular helper cells which support T cell differentiation |
| Immune blunting | Immune response after early MCV1 and after routine MCV2 in early MCV1 children versus after routine MCV1 and MCV2 in the short- and long-term |
|  | Role of high versus low maternal measles vaccine/infection induced immunity (overall and by age) on immune response after early MCV1 in elimination versus endemic settings |
|  | Relation between maternal measles vaccine/infection induced immunity and immune maturation level of young children |
|  | Modelling impact of blunting after routine MCV2 |
| **Abbreviation:** HIV=human immunodeficiency virus; MCV=measles-containing vaccine | |
| ^a^ Gaps were identified through an online survey during the plenary session of day 2. Seventy percent of the attendees responded (26 out of 37). Subsequently, the organizing committee thematized the gaps. Each theme was ranked according to the number of times a theme could be extracted from an attendee’s response. | |
